# Microarrays as Model Biosensor Platforms to Investigate the Structure and Affinity of Aptamers

**DOI:** 10.1155/2016/9718612

**Published:** 2016-03-03

**Authors:** Jennifer A. Martin, Yaroslav Chushak, Jorge L. Chávez, Joshua A. Hagen, Nancy Kelley-Loughnane

**Affiliations:** ^1^Human Effectiveness Directorate, 711 Human Performance Wing, Air Force Research Laboratory, Wright-Patterson Air Force Base, OH 45433, USA; ^2^UES, Inc., 4401 Dayton-Xenia Road, Dayton, OH 45432, USA; ^3^The Henry M. Jackson Foundation for the Advancement of Military Medicine, 6720A Rockledge Drive, Bethesda, MD 20817, USA

## Abstract

Immobilization of nucleic acid aptamer recognition elements selected free in solution onto the surface of biosensor platforms has proven challenging. This study investigated the binding of multiple aptamer/target pairs immobilized on a commercially available microarray as a model system mimicking biosensor applications. The results indicate a minimum distance (linker length) from the surface and thymine nucleobase linker provides reproducible binding across varying conditions. An indirect labeling method, where the target was labeled with a biotin followed by a brief Cy3-streptavidin incubation, provided a higher signal-to-noise ratio and over two orders of magnitude improvement in limit of detection, compared to direct Cy3-protein labeling. We also showed that the affinities of the aptamer/target interaction can change between direct and indirect labeling and conditions to optimize for the highest fluorescence intensity will increase the sensitivity of the assay but will not change the overall affinity. Additionally, some sequences which did not initially bind demonstrated binding when conditions were optimized. These results, in combination with studies demonstrating enhanced binding in nonselection buffers, provided insights into the structure and affinity of aptamers critical for biosensor applications and allowed for generalizations in starting conditions for researchers wishing to investigate aptamers on a microarray surface.

## 1. Introduction

Aptamers are oligonucleotide sequences that bind to a specific target molecule through noncovalent interactions [1, 2]. Aptamers have been implemented in a wide variety of applications, including analytical purification reagents, histological detection reagents, targeted delivery vehicles, and pharmaceuticals [3–6]. Moreover, aptamers have demonstrated particular promise as surface-immobilized biosensor recognition elements due to their stability, their ease of chemical modification, and high achievable densities when tethered to a platform. One critical issue associated with interfacing an aptamer with biosensor detection technology stems from the typical methodology used to initially identify the recognition elements. In SELEX (Systematic Evolution of Ligands by EXponential Enrichment), aptamers are generated by iterations of incubating oligonucleotides in free solution with targets followed by amplification over multiple rounds of selection [7, 8]. The solution-selected aptamer is then synthesized with a chemical tag for immobilization on a solid support for biosensor development. Previous studies have shown that the affinity of the aptamer for the target may be significantly diminished or destroyed after surface immobilization, potentially due to interactions with the surface and other probes denaturing or occluding the binding site, steric constraints that prevent proper aptamer folding and/or target interaction, or electrostatic repulsion of the target by the surface [9–11]. Therefore, understanding the performance and underlying structural features of an aptamer tethered to a surface is an important aspect influencing sensor design.

Microarray technology can be used to probe the functional characteristics of aptamers selected by solution-based SELEX in an immobilized format, mimicking the ultimate biosensing application. Microarrays indicate target binding by identifying locations of fluorescence from a fluorescently labeled target and correlating them with the position of known sequences on the array. Early attempts in microarray design suffered from challenges related to oligonucleotide deposition by spotting presynthesized probes onto an activated array surface, specifically in reproducibility when conditions (humidity, buffer, etc.) varied from batch to batch [12, 13]. Oligonucleotide spotting also rendered testing of thousands of sequences cost prohibitive due to the expense of synthesizing each probe individually prior to deposition onto the array. Current microarray technology based on synthesis from the array surface rather than oligonucleotide spotting produces high reproducibility and allows for up to 10^6^ completely custom 80 mer DNA sequences synthesized per array. The massively parallel nature of microarray testing drastically reduces the time and cost investments associated with assaying the functional characteristics of aptamers compared to traditional methods of individually synthesizing and purifying each sequence of interest. For example, a microarray experiment can be completed in less than one day, with data analysis requiring another 1-2 days. A single million feature array (Agilent 1×1M), similar to those used in the current study, at a price of ~$600 can assay up to a million sequences simultaneously, compared to ~$135 for one sequence HPLC purified from commercial vendors for traditional analysis.

One major challenge associated with applying microarray technology to aptamer studies lies in the fact that current protocols have been mostly developed for gene expression studies that function by direct hybridization of the fluorescent-tagged genomic fragments to cDNA on the microarray. This type of interaction forms an extremely stable duplex between the base pairs of the two strands, whereas aptamer/protein target interactions may heavily rely on one or a few noncovalent interactions. For example, the binding of the immunoglobulin E (IgE) aptamer has been shown to be completely destroyed following change of a single base within the aptamer sequence [11]. Moreover, the majority of dye systems used to label protein targets (“direct labeling”) for microarray work possess conjugated *π*-systems reported to interact with DNA directly [14]. This observation renders it challenging to differentiate between sequences which are binding the actual protein target and those that instead interact with the dye. Gold and coworkers reported using photoactive base substitutions to covalently cross-link proteins to the arrays, followed by stringent washing steps to remove nonspecific binders [15]. However, commercial microarray manufacturers do not currently offer these base substitutions in their array synthesis portfolio. A feasible method of biotinylating the protein followed by a short dye-labeled streptavidin incubation (indirect labeling) demonstrated less nonspecific binding compared to direct dye labeling [14, 16]. Furthermore, many parameters will affect the aptamer response that may not be as significant in cDNA hybridizations, including probe density, proximity of the binding site to the surface, immobilization orientation (5′ or 3′ end), and the buffer used in the studies [10, 17]. Therefore, protocols and methods for aptamers must be developed and optimized by the individual researcher accounting for challenges specific to the application.

As is the case with biosensor applications, the response of an aptamer in solution can be significantly different than when it is attached to a microarray surface [9]. Several studies report differences in aptamer affinity when comparing microarray and solution-based dissociation constant measurements [10, 11]. In most cases the affinities of these aptamers were significantly lower on the microarray than when tested in the solution-based conditions used during their selection [18]. Significant work has been performed by different groups in the use of aptamers for target detection in custom-made microarray settings. It has been confirmed that known DNA aptamers can be immobilized on a microarray and conditions to preserve their activity can be found, and the conditions used for these studies should mimic the conditions used during the initial solution-based selection process [12, 18]. Prior work has also demonstrated that extending the distance of the aptamers from the array surface enhances target binding; fluorescence intensity is proportional to the concentration of target added; and higher relative fluorescence values between probes on the same array indicate higher affinity interactions [11, 16]. Substantial work has also been presented describing the ability of microarrays to assay target binding after aptamer mutations, truncations, or changing conditions, but these studies have either not analyzed multiple aptamers binding multiple targets or not utilized specific conditions to analyze structural and functional information regarding target binding [11, 18, 19].

**Figure 1:**
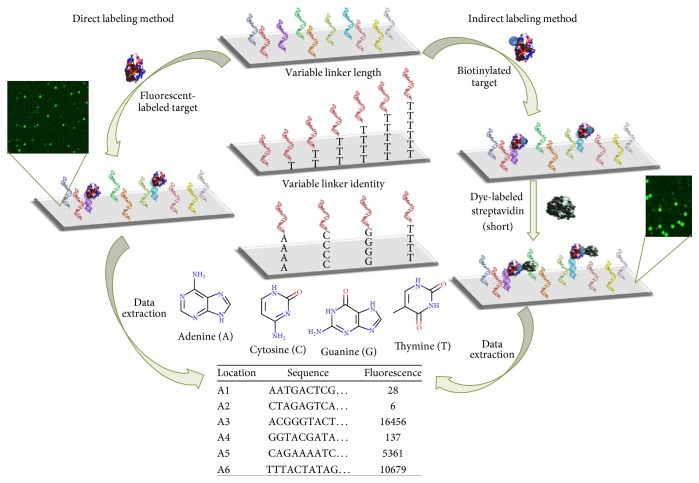
Diagram of variables investigated in the current experiment. Linker length: the distance from the microarray surface was varied; linker identity: nucleobase (adenine, cytosine, guanine, or thymine) was used to tether aptamers to the surface; direct labeling method: fluorescent reporter dye conjugated directly to the target molecule is incubated with aptamer microarray; indirect labeling method: biotinylated-target is added to microarray, followed by a short dye-streptavidin incubation with aptamer microarray. Following incubation, all microarrays were washed and imaged and the data was extracted in order to link fluorescence intensity location with aptamer sequence. Nucleobase structures were created using VIDA, part of OpenEye software.

The main goal of the current studies was to investigate microarrays as a model system to examine aptamer binding in an immobilized format mimicking that of biosensor applications. During the course of these studies, several different conditions were investigated related to the functional characteristics of multiple aptamers on custom microarrays. These enabled generalizations related to potential starting points for researchers wishing to utilize microarrays in future aptamer studies, as well as providing insights into specific aptamer/target interactions. Specifically, we investigated the effect of linker length with IgE as a control, and expanded the work to analyze the effect of the linker identity, dissociation constant (*K*_*d*_) under varying conditions, effect of the dye-to-protein ratio (D/P), and comparison of a direct and indirect labeling method [11]. The schematic in Figure 1 depicts a summary of the variables investigated using microarrays in the current study. In addition, the linker length and identity effects were extended to include several guanine-quartet (G-quartet) aptamers in relation to thrombin and dye binding. Furthermore, we utilized the known ability of G-quartets to bind *π*-conjugated dye systems to assess the structural characteristics of G-quartet aptamers under different conditions. This enables a correlation between the conditions and the aptamer structure without concern regarding whether the selected conditions may impart conformational changes on a protein target. Novel information on the structural and binding properties of the various aptamers used in this study was discovered, as well as experimental evidence backing the previously proposed structures of well reported thrombin and riboflavin (RB) aptamers. These studies collectively provide an indication that a minimal linker length may be necessary for reliable binding data and manipulation of the conditions can promote the interaction of weak binding sequences with their target, where binding was not previously observed. We propose that microarrays are an advantageous means to rapidly screen aptamer function under various conditions of interest, including different surface linkages, and buffer and salt conditions. The results described here indicate that microarrays can be used to probe the structural folding of aptamers, specifically whether they form a G-quartet, by a simple method of introducing a compatible dye to the system. This work is beneficial in the biosensor field, where transitioning a solution-selected aptamer to a surface-immobilized sensing platform has proven to be a nontrivial task.

## 2. Materials and Methods

### 2.1. Chemicals and Equipment

IgE (Fitzgerald), Illustra NAP-25 desalting columns and Cy3 Mono-Reactive Dye Pack (GE Healthcare), NanoDrop (Thermo Scientific), and nuclease free water (Gibco) were used. Microarray equipment is as follows: custom 8×15k and 1×1M DNA microarrays, 8×15k and 1×1M gasket slides, ozone barrier slides, hybridization chambers, scanner cassettes, hybridization oven, and High-resolution Microarray Scanner (all Agilent), Slide rack and wash dishes (Shandon), and Kimtech polypropylene wipes (Kimberly-Clark). FluoReporter Mini-Biotin-XX Protein Labeling Kit (Invitrogen), Streptavidin-Cy3 Conjugate and HABA/Avidin Reagent (Sigma), and dialysis cassettes (3.5k MWCO, Thermo Scientific) were also used.

### 2.2. Buffers

Buffers are as follows:

 binding [PBSMTB]: 1x PBS (8.1 mM Na_2_HPO_4_, 1.1 mM KH_2_PO_4_, 2.7 mM KCl, 137 mM NaCl, pH 7.4) + 1 mM MgCl_2_ + 0.1% Tween-20 and 1% BSA;
 washing [PBSM]: 1x PBS (8.1 mM Na_2_HPO_4_, 1.1 mM KH_2_PO_4_, 2.7 mM KCl, 137 mM NaCl, pH 7.4) + 1 mM MgCl_2_;
 rinse [R]: 1/4 dilution of PBSM and nuclease free water;
 IgE binding buffer without K^+^: (8.1 mM Na_2_HPO_4_, 137 mM NaCl, pH 7.4) + 1 mM MgCl_2_ + 0.1% Tween-20 and 1% BSA;
 washing: 8.1 mM Na_2_HPO_4_, 137 mM NaCl, pH 7.4 + 1 mM MgCl_2_;
 RB Wash Buffer: 20 mM HEPES, 300 mM KCl, 5 mM MgCl_2_;
 RB binding buffer: 20 mM HEPES, 300 mM KCl, 5 mM MgCl_2_ + 0.1% Tween-20 + 1% BSA.

### 2.3. Cy3-Labeling of IgE and Thrombin

IgE was diluted to 1 mg/mL with 0.1 M sodium carbonate buffer (pH = 9.3). One mL of 1 mg/mL IgE was added to one Cy3 dye pack and incubated 30 min. Free dye was separated from IgE-bound dye by purification with a desalting column. The product was then injected into dialysis cassettes for two separate 2 h incubations with fresh 400 mL PBSMTB at room temperature and then overnight with 400 mL fresh PBSMTB at 4°C. Dye-to-protein ratio (D/P) was calculated using the manufacturers' instructions with the aid of NanoDrop UV/Vis detection.

Thrombin was labeled with Cy3 in a similar manner to IgE. Thrombin was incubated for 30 minutes after dilution in 1 mL 0.1 M Na_2_CO_3_ buffer (pH = 9.3) to 1 mg/mL. The product was purified with a Texas Red protein labeling size exclusion column (Molecular Probes) to yield a D/P = 0.8 using the manufacturer's instructions via NanoDrop UV/Vis detection.

### 2.4. 8×15k Microarray Design and Methodology

Aptamers were synthesized on custom 8×15k microarray slides in replicates ranging from 5 to 15 by Agilent Technologies. Blocking with PBSMTB was performed on the DNA microarray loaded into a 50 mL conical tube for 1 h at room temperature. Slides were disassembled in PBSM buffer and rinsed for 5 min at room temperature using a stir plate. The slides were quickly edge-tapped to remove excess buffer. Seventy *μ*L protein (variable concentrations) in PBSMTB was loaded onto the gasket slide then incubated with each of the 8 DNA arrays for 2 hrs at 20°C in a hybridization chamber/hybridization oven. Slides were disassembled in PBSMTB buffer and washed for 3 min in a separate PBSM buffer with the slide rack and stir plate; then the slide rack was transferred to 1/4 PBSM buffer/water for 1 min using a stir plate. Slides in the slide rack were dipped in nuclease free water to remove any remaining salt and washed for 1 min with stir plate. The rack was slowly withdrawn from the water to promote a drier surface; the back of the slide was wiped with ethanol and then placed in a 50 mL conical tube with a polypropylene wipe at the bottom and centrifuged at 4150 rpm for 3 min. The microarray was loaded into a scanner cassette and covered with an ozone barrier slide before scanning.

### 2.5. 1×1M Microarray

The basic protocol was the same for the 1×1M arrays as for the 8×15k arrays described above. The only difference in methodology was that the gasket slide held a 750 *μ*L volume instead of 70 *μ*L.

### 2.6. Indirect Labeling Method IgE Biotinylation

Biotinylation of IgE was carried out according to the manufacturer's instructions and purified using the provided spin column. Briefly, 200 *μ*L 1 mg/mL IgE was mixed with 20 *μ*L 1 M NaHCO_3_, pH = 8.32. The biotin-XX material was dissolved in 200 *μ*L nuclease free water and 2 *μ*L solution was transferred to the protein/NaHCO_3_ mixture. The solution was stirred for 1 hour at room temperature and then added to the prepared spin column. The biotin/protein ratio was calculated to be 0.6 according to the HABA/Avidin test.

### 2.7. 8×15k and 1×1M Microarrays (Indirect Labeling Method)

The 8×15k and 1×1M microarrays were analyzed in the same manner as the direct labeling method with some exceptions. Following incubation with biotin-IgE, slides were disassembled in PBSMTB buffer and rinsed for 3 min on a shaker. Slides were transferred to PBSM buffer for 3 min also on a shaker. Then 0.5, 10, or 500 nM Cy3-streptavidin (Cy3-SA) was loaded into the gaskets and incubated for 2 min. The washing steps were the same as the direct labeling method.

### 2.8. 8×15k Arrays with Variable Targets

The basic protocol was similar to the 8×15k direct labeling protocol, except that the samples and concentrations were variable: 100 nM samples: Cy3-IgE D/P = 2.4, 0.41, and 0.28, Cy5, Cy3-thrombin; 50 nM: Cy3; 10 nM: Cy3-streptavidin. When the buffers were varied, the arrays were incubated in the buffer of interest before sample loading, and slides were disassembled in water then quickly transferred (3 seconds) to PBSM for the subsequent washing steps.

### 2.9. Data Analysis

The arrays were scanned using Agilent Scan Control software, generating TIFF images using 20-bit imaging at 3 *μ*m (for 1×1M arrays) or 5 *μ*m (8×15k arrays). Data was extracted using Agilent Feature Extraction software version 10.7.3.1. Code was written in Perl to provide mean fluorescence intensity and standard deviation of the replicates for each probe investigated.

## 3. Results and Discussion

### 3.1. Linker Length Testing Using IgE

**Figure 2:**
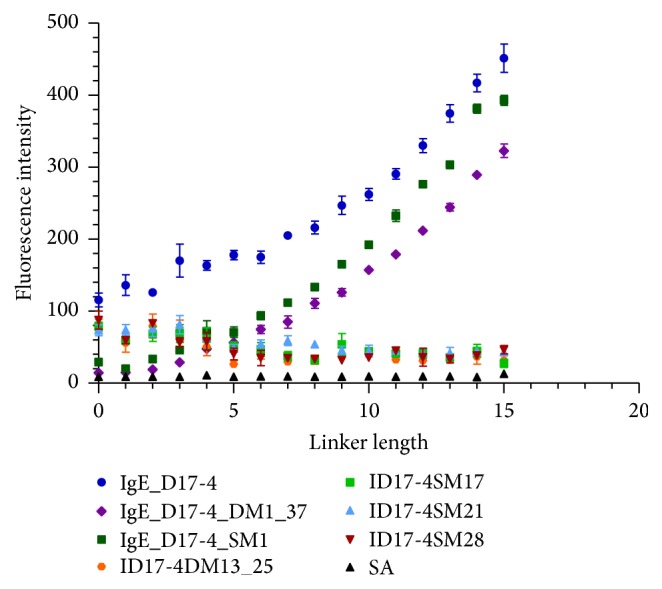
Response of different T linker sequences to 100 nM Cy3-IgE in PBSMTB buffer. Error bars represent SEM of raw fluorescence intensity values for 5–15 replicates of each sequence.

The current study tested the T linker response for the initial IgE binding aptamer, IgE_17-4, at 100 nM Cy3-IgE, and observed an increase in fluorescence response from linker length 0–15 T (Figure 2; [20]). This agrees with prior studies demonstrating that increasing the (thymine) T-linker length separating the aptamer from the surface of the microarray generated an increase in sensor response [11]. The initial IgE sequence, IgE_17-4, was mutated by Katilius et al. on a microarray in a series of single or double mutations to generate new sequences which retained target binding capabilities [11]. The current study showed that positive mutants IgE_D17-4_DM1_37 and IgE_D17-4_SM1 demonstrated similar trends but displayed lower fluorescence intensity values at shorter linker lengths than IgE_17-4 (See Table S1 for a description of sequences used in the current study) (see Supplementary Material available online at http://dx.doi.org/10.1155/2016/9718612). As the linker length was extended, the two mutants produced fluorescence intensity values approaching that of the initial IgE aptamer (Figure 2). Also, several mutations that were shown in the Katilius work to reduce binding (ID17-4DM13_25, ID17-4SM17, ID17-4SM21, and ID17-4SM28) demonstrated fluorescence intensities similar to the initial aptamer and higher than the two binding mutants (SM1 and DM1_37) at short linker lengths [11]. However, the reduced binding mutants decreased in fluorescence intensity as the linker length was increased. Potentially the weak binding sequences are held in a vertical orientation at short linker lengths which primarily exposes the target binding site, but the increased flexibility at higher linker lengths allows for a broader range of orientations or inter/intramolecular and surface interactions which cannot be compensated for because of the poor binding affinity. Nonspecific binding between the target and aptamer backbone or surface may also dominate at short linker lengths. This trend held true across all eight concentrations tested; examples of 25 nM and 200 nM are included, as well as a 1×1M feature array (Figures S1-3). A nonbinding streptavidin aptamer mutant (SA) showed constant low background fluorescence intensity across all linker lengths (Figure 2). The binding of IgE to the negative mutants was always higher than SA binding, demonstrating that protein binding was reduced but not completely eliminated.

### 3.2. Linker Identity Testing Using IgE

**Figure 3:**
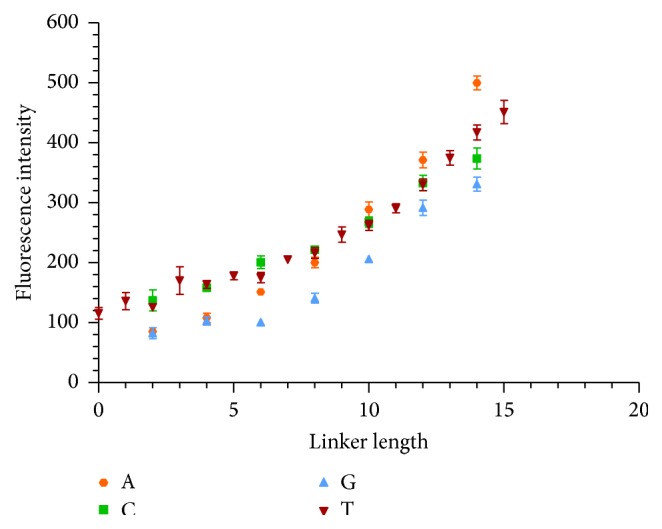
Response of IgE_D17-4 aptamer to 100 nM Cy3-IgE using different nucleobase linkers in PBSMTB buffer. Error bars represent SEM of raw fluorescence intensity values for six replicates of each sequence.

The effect of the linker identity was also examined as a function of linker length for the aptamer IgE_17-4 by implementing A, C, G, and T nucleobase linkers.  Figure 3 shows the results of changing the base identity (A, C, G, and T linker) on fluorescence intensity at 100 nM Cy3-IgE. T and C bases perform similarly at short linker lengths, but T outperforms C as the length increases. The trend holds true for 25 nM and 200 nM IgE concentrations as well and at 100 nM Cy3-IgE on a 1×1M feature array (Figures S4-6). A base demonstrates lower fluorescence intensity than T at short lengths and then begins to outperform T at longer lengths, while G consistently shows the lowest fluorescence intensity. This result is reasonable considering that the C and G bases have potential to form more complex structures, potentially reducing the expected distance from the surface or altering the binding properties of the sequence. In summary, the results show that different bases will provide varying signals, and the T base is a consistently high performer, but may be outperformed by A base at longer linker lengths.

### 3.3. Effect of Linker Length and Identity on IgE Aptamer/Target Affinity

The next question investigated was how linker length and identity will affect the actual aptamer/target binding affinity, measured by dissociation constant (*K*_*d*_). Further information on methods and discussion related to generation and processing of the binding curves can be found in the Supporting Information. In the current study, the aptamer response was analyzed for each linker length and identity at Cy3-IgE concentrations up to 200 nM. The fluorescence response increased with an increase in linker length for the original IgE_17-4 aptamer and the positive mutants but was close to zero for negative controls after background subtraction, indicating nonspecific binding for the negative sequences (Figure S7). The microarray results suggested that the affinity was not significantly different between T linker (*K*_*d*_ = 66.3 ± 3.1 nM) and A linker (*K*_*d*_ = 63.7 ± 1.7 nM) or C linker (61.8 ± 3.8 nM), but *K*_*d*_ = 86.5 ± 8.1 nM for G is statistically significant from T (*p* = 0.0239, 95% CI). Binding constants were also determined for the mutants SM1 (79.9 ± 3.8 nM) and DM1_37 (82.3 ± 6.5 nM), both of which are significantly (*p* = 0.020 and *p* = 0.0497, 95% CI) higher than IgE_17-4 T linker (Figure S7). The affinity of the interaction was not improved by increasing the linker length, despite the gains in sensitivity from the higher overall fluorescence intensity.

The average *K*_*d*_ for the IgE_17-4 T linker (66.3 ± 3.1 nM) differs from three separate binding assays where the aptamer was not immobilized to a surface: nitrocellulose (*K*_*d*_ = 9 nM), surface plasmon resonance (SPR; *K*_*d*_ = 4.7 nM), and fluorescence anisotropy (*K*_*d*_ = 15 ± 4 nM) [11, 20]. This is not unexpected since past investigations have generally shown that surface immobilization of an aptamer selected in free solution may result in diminished affinity for the target [10, 17, 18]. However, the microarray affinity from the current study does agree with an SPR-based study where the IgE aptamer was immobilized on the surface, demonstrating *K*_*d*_ = 74.8 nM [21]. The affinity reported in the current study also varies from prior microarray studies estimating *K*_*d*_ > 100 nM [11, 19]. We suspect that the differences in dye-to-protein (D/P) ratios, incubation time and temperature, buffer conditions, and background subtraction data processing step contribute to this discrepancy between microarray studies [11, 19].

### 3.4. Effect of Linker Length and Identity on Fit Quality of IgE Aptamer/Target *K*_*d*_ Curves

**Figure 4:**
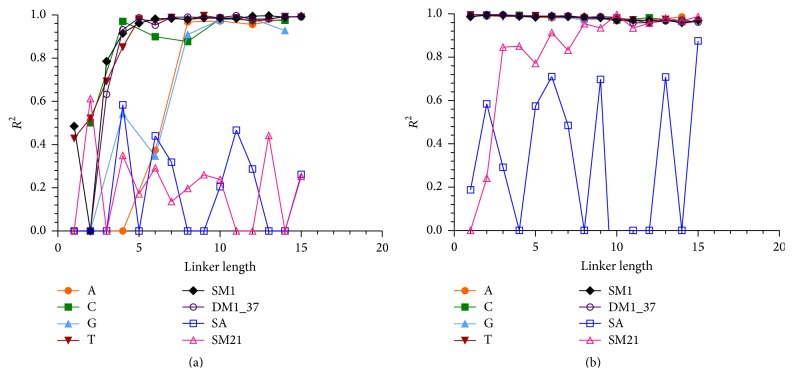
Comparison of coefficient of determination (*R* ^2^) versus linker length for (a) direct labeling method and (b) indirect labeling method. Curves were fit to a one site specific binding with Hill slope model using GraphPad Prism. A (circle), C (square), G (triangle), and T (downward triangle) are the linkers used for IgE_17-4 aptamer. The remaining sequences (SM1, DM1_37, SA, and SM21) all possessed T linkers.

Analyzing the binding affinity of the aptamer/target interaction in relation to the quality of the fit of the data to the binding model provides additional information on optimal conditions. At short linker lengths, the binding curves did not fit the data set, or the error was high in the *K*_*d*_ estimate. A plot of the coefficients of determination (*R* ^2^) across linker lengths shows the improvement in fit as linker length increases (Figure 4(a)). All of the IgE binders have poor data fits at short lengths and then improve until a plateau is reached close to *R* ^2^ = 1.0. This effect may result from a strong dependence on nonspecific binding at short lengths and/or because the rigidity of the linkage to the surface does not allow for the aptamer to accommodate target binding. This implies that a minimal linker length (~10 nucleobases) should be applied to achieve high-quality binding data. Increasing the length of the linker past this point will increase the sensitivity (fluorescence intensity) of the response by generating higher overall signal but will not affect binding. Nonbinding sequences (SA and SM21) either were not able to produce a curve fit at all (*R* ^2^ = 0) or produced a poor curve fit which never reached acceptable (*R* ^2^ > 0.95) levels. As a whole, the binding studies show that both the linker length and identity can influence aptamer binding under the conditions of this study.

### 3.5. Effect of Labeling Method on IgE Aptamer/Target Binding

**Figure 5:**
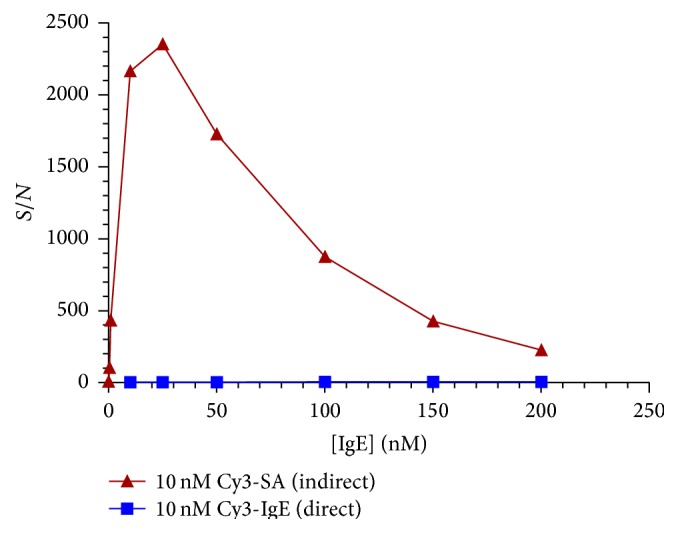
Signal-to-noise (S/N) comparison using direct and indirect dye labeling under different conditions for T_10_ IgE_17-4. Biotin-IgE and Cy3-IgE concentrations were 10 nM.

The method used to report a binding event from the protein target is another important facet of microarray studies. Most dye systems used to label proteins for microarray work possess conjugated *π*-systems reported to interact with DNA directly, which may manifest in a nonspecific binding component [14]. A feasible method of biotinylating the protein followed by a short dye-labeled streptavidin incubation has been shown to demonstrate less nonspecific binding compared to a direct dye labeling [14, 16]. We hypothesized that the lower background expected in this “indirect” protein labeling strategy may increase the signal to noise ratio (S/N) and thereby lower limits of detection (LOD) compared to the direct labeling method. The average fluorescence intensity of the T_10_ IgE_17-4 aptamer was divided by the average fluorescence intensity for the SA to determine the signal to noise ratio for each set of conditions. The S/N of the indirect method is a maximum of over 650X higher than the direct labeling method (Figure 5). At [biotin-IgE] = 10 nM, [Cy3-SA] = 10 nM, the LOD was improved to 4 pM from 500 pM with [Cy3-IgE] = 10 nM in the direct method. Comparison of the images of the arrays with only 10 nM Cy3-streptavidin or 10 nM biotin-IgE + 10 nM Cy3-streptavidin shows that the fluorescence is a result of IgE binding rather than a nonspecific interaction with streptavidin or Cy3 (Figure S8). These results show that the indirect labeling method has enhanced signal and lower nonspecific binding compared to the direct labeling method.

### 3.6. Effect of Indirect Labeling Method on IgE Aptamer/Target Affinity and *K*_*d*_ Curve Fit

Binding curves were also generated for IgE sequences using the indirect labeling method with [biotin-IgE] = 10 nM, [Cy3-SA] = 10 nM (Figure S9). The *K*_*d*_ for IgE_17-4 T linker was 29.5 ± 0.7 nM, which was approximately half that reported using the direct labeling method. All other nucleobase linkers were close in affinity to the T linker with A linker *K*_*d*_ = 30.9 ± 0.4 nM, C linker *K*_*d*_ = 30.8 ± 0.1 nM, and G linker *K*_*d*_ = 29.5 ± 0.8 nM. The fact that the G linker displayed weaker affinity than the other nucleobase linkers using the direct labeling method but all linkers were similar using the indirect labeling method may be due to minimization of the fluorescence reporter interaction with the surface using the indirect labeling method. Furthermore, Cy3 may occlude the binding site of IgE using the direct labeling method, where the biotinylation performed for the indirect labeling method did not interfere with binding. The affinities of the IgE_SM1 and IgE_DM1_37 sequences also aligned with *K*_*d*_ = 28.8 ± 0.6 nM and *K*_*d*_ = 29.9 ± 0.8 nM, respectively. Additionally, binding was observed for aptamer IgE_17-1 (Figure S9I; *K*_*d*_ = 73.4 ± 5.1 nM) using the indirect labeling method where it was not evident during direct labeling (Figure S7I). This agrees with the solution-based nitrocellulose assay performed following the original selection for IgE_17-1 reporting a *K*_*d*_ = 82 nM [20]. The trends of coefficient of determination are different from those of the direct binding method (Figure 4(a)) in that there does not appear to be a strong dependence on linker length for the indirect labeling method (Figure 4(b)). All of the IgE binders are close to *R* ^2^ = 1.0 at all linker lengths, while the nonbinder fits are more variable. The model could not accurately fit SA or SM21 binding data, but it appears that SM21 has some weak binding activity likely still in the linear range (Figure S9H, inset). Using the indirect labeling method resulted in higher affinity binding with less dependence on linker identity and length than direct labeling under the conditions of this experiment.

### 3.7. Effect of Labeling Method on S/N for IgE Aptamer/Target Binding

**Figure 6:**
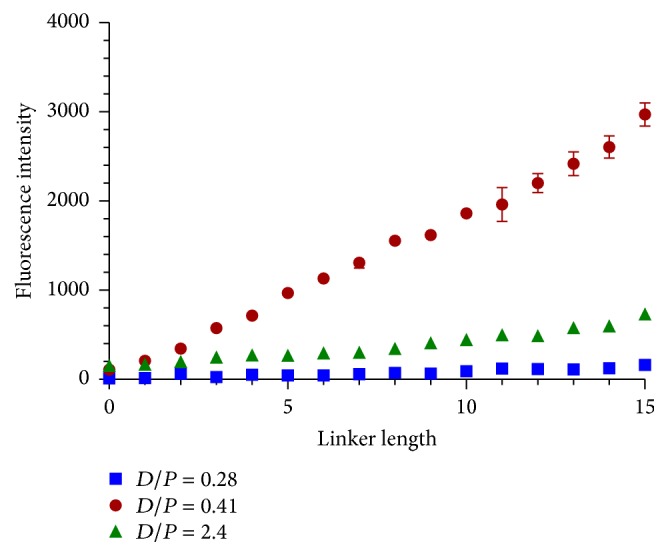
Variation of dye-to-protein (D/P) ratio of 100 nM Cy3-IgE for direct labeling method. Error bars represent SEM for six replicates of IgE_17-4 aptamer with T linker.

The D/P of the direct labeling method was investigated next to deduce whether the cause of the enhanced binding in the indirect method was due to the labeling of IgE. Previous work has shown that an excess of dye can occlude the binding site of the target or diminish fluorescence yield by resonance energy transfer [9, 22]. Therefore, the D/P = 2.4 used in the experiments described above was compared to D/P = 0.41 and 0.28. At 100 nM IgE concentration, Figure 6 shows that reducing the D/P from 2.4 to 0.41 provides the highest fluorescence intensity of the ratios tested, while further reduction to 0.28 shows the lowest fluorescence intensity. This demonstrates the tradeoff between decreasing D/P to allow for more binding and the lower fluorescence intensities that result at some cutoff point. In this experiment, the S/N values were 186 for D/P = 0.41, 5.3 for D/P = 2.4, and 4.9 for D/P = 0.28 versus 875 for 100 nM biotin-IgE with 10 nM Cy3-SA. Further optimization of the D/P or biotin-to-protein ratio may improve the S/N for the direct and indirect labeling methods, respectively. This shows that S/N can be improved by adjusting D/P on a protein target using a direct detection method, but it is still not as sensitive as the indirect labeling method. Binding of aptamer IgE17_1 was also restored at D/P = 0.41, where it was not observed at D/P = 2.4, illustrating that the binding properties of an aptamer are influenced by D/P.

### 3.8. Effect of Linker Length and Identity for Cy3-Thrombin Binding G-Quartet Aptamers

**Figure 7:**
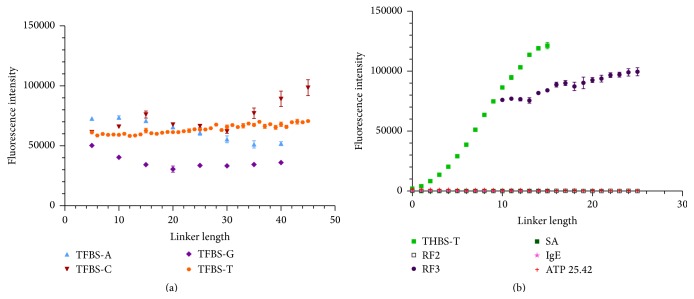
Binding of Cy3-thrombin to various sequences. (a) Binding of Cy3-thrombin to TFBS aptamer using different nucleobase linkers and lengths; (b) Binding of Cy3-thrombin to T linker variants of THBS (thrombin binding sequence and reported G-quartet former), RF2 (riboflavin aptamer and reported G-quartet former), RF3 (riboflavin aptamer and reported G-quartet former), SA (streptavidin aptamer mutant and non-G-quartet former), IgE_D17-4 (IgE aptamer and non-G-quartet former), and ATP 25.42 (ATP aptamer initially reported to form G-quartet, but later determined to form pseudoknot structure). Error bars represent SEM of raw fluorescence intensity values for 5–15 replicates of each sequence. Linker identity was T unless otherwise specified.

We next examined the interaction of Cy3-thrombin with aptamers reported to form G-quartet structures. The binding of Cy3-thrombin with the 15-mer thrombin aptamer TFBS, the 29-mer thrombin G-quartet aptamer THBS, the 2-tiered G-quartet riboflavin aptamer RF2, and the 3-tiered G-quartet riboflavin aptamer RF3 was investigated [23–25]. The linker length and identity also affect thrombin binding to TFBS, though in a different manner than IgE binding IgE_17-4 (Figure 7(a)). Similar to the IgE profile, T linker shows a consistent fluorescence increase to long linker lengths, and G linker is regularly the worst performer across all linker lengths. However, fluorescence signal actually decreases with increasing length for A linker where it increased for all base linkers in IgE/IgE_17-4 binding. A is initially the best performer for TFBS/thrombin and then decreases at longer lengths in contrast to IgE/IgE_17-4 results, while C results are variable. Structurally this may indicate that certain bases included in the linker may interact with the aptamer sequence and affect target binding. This shows that the performance will vary with the linker ID depending on the aptamer target pair, and the effects should be investigated prior to implementation. The effects of thrombin binding THBS (Figure 7(b)) are much more dramatic across linker lengths, as the fluorescence intensity increases from slightly above background at T_0_ to two orders of magnitude increase at T_15_. This agrees with previous reports that contend that the THBS aptamer consists of a structure that requires extra spatial mobility to fold into an active conformation due to the longer duplex between the 5′ and 3′ terminus and has reduced stability in the G-quartet compared to TFBS [24]. Also, thrombin demonstrates enhanced binding to RF3 compared to TFBS, but no binding to the 2-tiered RF2 aptamer (Figure 7(b)). Binding of thrombin to RF3 is not unexpected due to studies that showed that the core G-quartet region of TFBS (similar in RF3) is necessary for binding, while changes to the identities of the loop sequences are tolerated with different levels of success [16, 26, 27]. The lack of RF2 binding is expected as well due to reports indicating that RF2 is less stable than RF3 in a Na^+^ based buffer and requires a higher K^+^ content for structural stability to fold into the G-quartet structure [25]. Thrombin did not bind significantly to SA, IgE_17-4, or an ATP aptamer (ATP 25.42) initially reported to fold into a G-quartet but later shown to actually form a pseudoknot instead (Figure 7(b); [28, 29]).

### 3.9. Effect of Linker Length and Identity for Cyanine Dye Interaction with G-Quartet Aptamers

**Figure 8:**
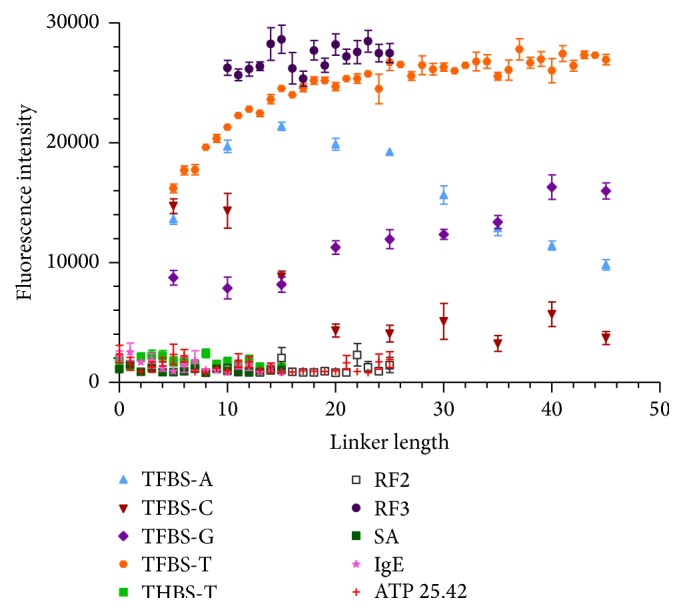
Binding of Cy3 dye in PBSMTB buffer to a variety of G-quartet and non-G-quartet aptamer sequences. Error bars represent SEM of raw fluorescence intensity values for 5–15 replicates of each sequence. Linker identity was T unless otherwise specified.

Previous research has shown that fluorescent dyes can intercalate between G-quartets [30–32]. This property was exploited to propose that microarrays can be used to determine whether a sequence folds into a G-quartet structure and may also be used to study structural properties of G-quartet aptamers under different conditions by adding dye directly to the array.  Figure 8 shows a strong response for TFBS with Cy3 dye, which varies depending on linker identity and length. Contrary to the interaction between Cy3-thrombin and TFBS, G linker increases in intensity as linker length increases, presumably due to more G-quartets forming within the linker. C is the worst performer at long lengths, potentially due to the C bases pairing with some of the G's in the G-quartet aptamer portion, altering the binding conformation of TFBS. A linker demonstrates a trend similar to that of thrombin binding and T is the best performer, increasing and then plateauing. The stronger dependence of Cy3 binding TFBS as T linker length is increased as compared to thrombin binding may be due to the increased space required to compensate for electrostatic repulsion of the sulfate groups of Cy3 by the DNA backbone. The larger size of thrombin may also sterically limit the benefits of extending the aptamer from the array surface. Aptamer RF3 displayed enhanced fluorescence when interacting with Cy3 dye, while RF2 did not demonstrate fluorescence above background, similar to the interaction with thrombin. THBS fluorescence was slightly above SA background but drastically diminished compared to the THBS/thrombin interaction. This may be due to competition between K^+^ and Cy3 for binding or by K^+^ inducing a conformational change which diminishes Cy3 binding. Cy5 dye also interacts with TFBS, following a trend very similar to Cy3 binding in PBSMTB (Figure S10). This work indicates that cyanine dyes can be used to probe whether aptamers fold into a G-quartet on a microarray surface and to glean structural information related to the binding parameters. Based on the performance of all the aptamer/target pairs studied in this work, T base is recommended as a safe starting point for sensor development applications.

### 3.10. Effect of Buffer Conditions on Cyanine Dye Interaction with G-Quartet Aptamers

**Figure 9:**
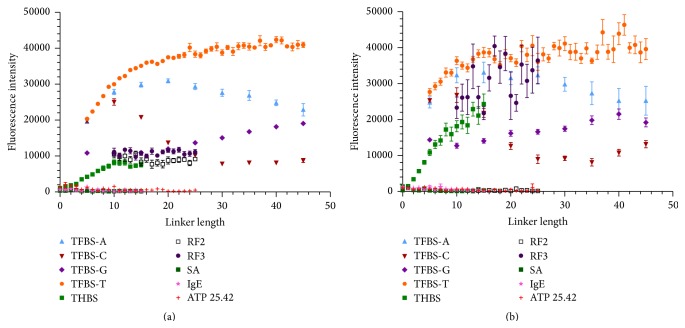
Binding of Cy3 dye to a variety of G-quartet and non-G-quartet aptamer sequences using (a) initial RF aptamer selection buffer; (b) PBSMTB without K^+^. Error bars represent SEM of raw fluorescence intensity values for 5–15 replicates of each sequence. Linker identity was T unless otherwise specified.

In order to further probe the structural features of the G-quartet aptamers, the sequences were exposed to various buffer conditions with Cy3 target.  Figure 8 illustrated that RF2 did not bind Cy3 dye in PBSMTB buffer, which was postulated to be due to RF2 decreased binding in Na^+^ buffers and high dependence on K^+^ ion. The same experiment was then repeated to assess RF2 binding in the original selection buffer, containing no Na^+^ and high (300 mM) K^+^. RF2 binding was equivalent to RF3 binding under these conditions, indicating that the RF buffer allowed RF2 to form a properly folded G-quartet structure (Figure 9(a)). However, RF3 binding was significantly diminished in the original RF buffer compared to PBS-based buffer (Figure 7(b)) where it demonstrated higher fluorescence intensity than TFBS. THBS binding was also enhanced in the Cy3-targeted RF buffer compared to PBSMTB (Figure 9(a)). This led to questions on the role of the K^+^ ion for these different G-quartet aptamers binding Cy3 dye. When PBS buffer was used without any K^+^ component, RF2 binding was eliminated and the binding of all probes had higher error for each measurement (Figure 9(b)). TFBS binding was similar between RF and PBS (no K^+^) buffers, while THBS and RF3 binding were enhanced in the PBS (no K^+^) buffer, indicating a minimal dependence on K^+^ for G-quartet binding under the experimental conditions. This shows that certain sequences have different sensitivities to buffer composition, and some aptamers may show enhanced binding in buffers other than the original selection buffer. Microarrays afford the researcher the opportunity to rapidly study various conditions in parallel to optimize the performance for a biosensor application.

## 4. Conclusions

The current research investigated the binding and structural properties of aptamers immobilized on a microarray surface which were initially selected by solution-based SELEX. The microarray format was specifically chosen as a platform which would allow for the study of aptamers in an immobilized format, similar to most biosensing applications. As a result of this study, we were able to make generalizations regarding conditions researchers may wish to use as a starting point for future microarray aptamer studies. This is particularly interesting because most microarray protocols provided by commercial vendors are for cDNA hybridizations which are much stronger than the noncovalent interactions experienced by aptamer/target binding. Therefore, to date, most aptamer microarray procedures are optimized from scratch for each aptamer target pair. In the current study, we show that careful attention should be paid to the identity of the linker used, as well as to the distance of the aptamer from the microarray surface (linker length). T linker appears to be a consistent choice for linker across different types of aptamer/target pairs, while G linker was the weakest performer, perhaps due to the formation of intralinker secondary structure as the length increases. We also show that a minimal linker length may be required to produce reliable binding data and that extending the length past approximately 10 nucleobases increases the sensitivity of the response but does not affect the affinity. Furthermore, an indirect labeling method was shown to have a higher S/N than the direct labeling method, potentially due in part to minimized interaction of the fluorescent reporter with the microarray surface. The S/N can be modulated to some degree by changing the D/P of the dye linked with target but did not reach the high levels attained by the indirect labeling method. Collectively, this work describes some baseline conditions which may reduce the optimization time of future studies. A further result of these studies was to report novel information on specific aptamer/target interactions. As an example, the indirect binding method demonstrated binding for the weak binding IgE_17-1 and ID17-4SM21 sequences, which was not observed using the direct binding method. This work also experimentally indicated that theoretical propositions of thrombin and riboflavin aptamer structures may be accurate under different conditions. In addition, we show that microarrays can be used as a simple method for determining whether an aptamer forms a G-quartet structure by adding a compatible dye to the surface. This method is simpler than previous methods that require sequence-specific assignments to determine aptamer structure and is complementary to the recently reported benefits of structural screening by a combination of imino proton NMR and 2D NOESY [33].

The simplicity of the microarray experiments and rapid timeframe from lab work to data analysis promote the use of the technology for screening the effect of a variety of conditions on aptamer binding to optimize the interaction. In addition to those assayed in this work, the microarray could be instrumental in rapidly assessing aptamer candidates for function at enhanced temperature, such as those mimicking physiological conditions. Microarray technology could also be applied to rapidly assess a multitude of aptamers for cross-reactivity to physiologically relevant targets as would be anticipated to have importance in point-of-care applications. This work demonstrated design considerations and benefits of microarray technology toward an improved understanding of the aptamer structure/function relationship aimed at effectively integrating solution-selected aptamers onto biosensing platforms.

## Supplementary Material

Supplementary Material includes a summary of sequences investigated in this study, and various concentration and replicate examples in addition to the conditions described in the manuscript. The binding curves and experimental details for direct and indirect labeling methods are included as well.
