# Supplementary material for: Microarrays as Model Biosensor Platforms to Investigate the Structure and Affinity of Aptamers
Source: J Nucleic Acids. 2016 Mar 3;2016:9718612. doi: 10.1155/2016/9718612 (PMC4794571; doi:10.1155/2016/9718612)
Supplement: Supplementary file 1 — Supplementary Material includes a summary of sequences investigated in this study, and various concentration and replicate examples in addition to the conditions described in the manuscript. The binding curves and experimental details for direct and indirect labeling methods are included as well. [file 9718612.f1.docx]

| **Table S1.** Characteristics of Sequences Studied on Microarray | | | |
| --- | --- | --- | --- |
| Name | Sequence | Target | G-Quartet |
| TFBS | GGT TGG TGT GGT TGG | Thrombin (+) | Yes |
| THBS | AGT CCG TGG TAG GGC AGG TTG GGG TGA CT | Thrombin (+) | Yes |
| ID17-4DM13_25 | GGG GCA CGT TTA GCC GTC CCT CCT TGT GGC GTG CCC C | IgE (-) | No |
| ID17-4SM17 | GGG GCA CGT TTA TCC GAC CCT CCT AGT GGC GTG CCC C | IgE (-) | No |
| ID17-4SM21 | GGG GCA CGT TTA TCC GTC CCG CCT AGT GGC GTG CCC C | IgE (-) | No |
| ID17-4SM28 | GGG GCA CGT TTA TCC GTC CCT CCT AGT AGC GTG CCC C | IgE (-) | No |
| IgE_D17-4_DM1_37 | AGG GCA CGT TTA TCC GTC CCT CCT AGT GGC GTG CCC T | IgE (+) | No |
| IgE_D17-4_SM1 | TGG GCA CGT TTA TCC GTC CCT CCT AGT GGC GTG CCC C | IgE (+) | No |
| IgE_D17-4 | GGG GCA CGT TTA TCC GTC CCT CCT AGT GGC GTG CCC C | IgE (+) | No |
| IgE_D17-1 | CA CGT TTA TCC GTC CCT CCT AGT GGC GTG | IgE (+) | No |
| SA | TCT GTG AGA CGA CGC ACC GGT CGC AGG TTT TGT CTC ACA G | Streptavidin (-) | No |
| RF2 | GGA ACG ACG GTG GTG GAG GAG ATC GTT CC | Riboflavin (+) | Yes |
| RF3 | GGA ACG ACG GGT GGG TGG GAG GGA GAT CGT TCC | Riboflavin (+) | Yes |
| ATP 25.42 | CCT GGG GGA GTA TTG CGG AGG AAG G | ATP (+) | No |

DM and SM are double mutation, and single mutation, respectively. Red text indicates the location of the altered nucleobase relative to the original aptamer IgE_D17-4. Target column illustrates the target selection the aptamer was identified from, and whether the sequence binding (+) or non-binding (-). The G-Quartet column reports whether the structure of the sequence is known to fold into the G-Quartet conformation.


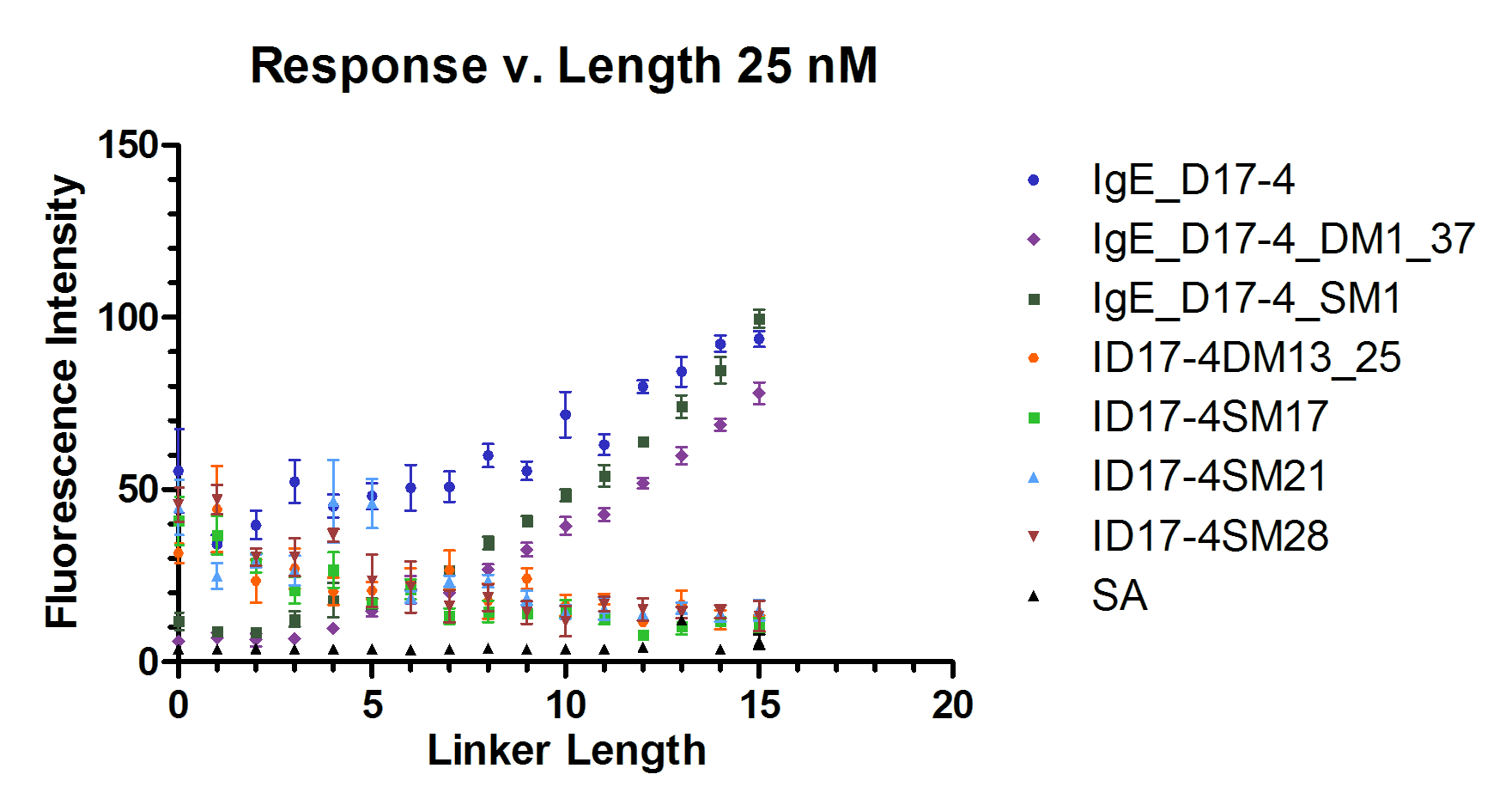


**Figure S1.** Response of different T linker sequences to 25 nM Cy3-IgE in PBSMTB buffer. Error bars represent SEM of raw fluorescence intensity values for 5-15 replicates of each sequence.


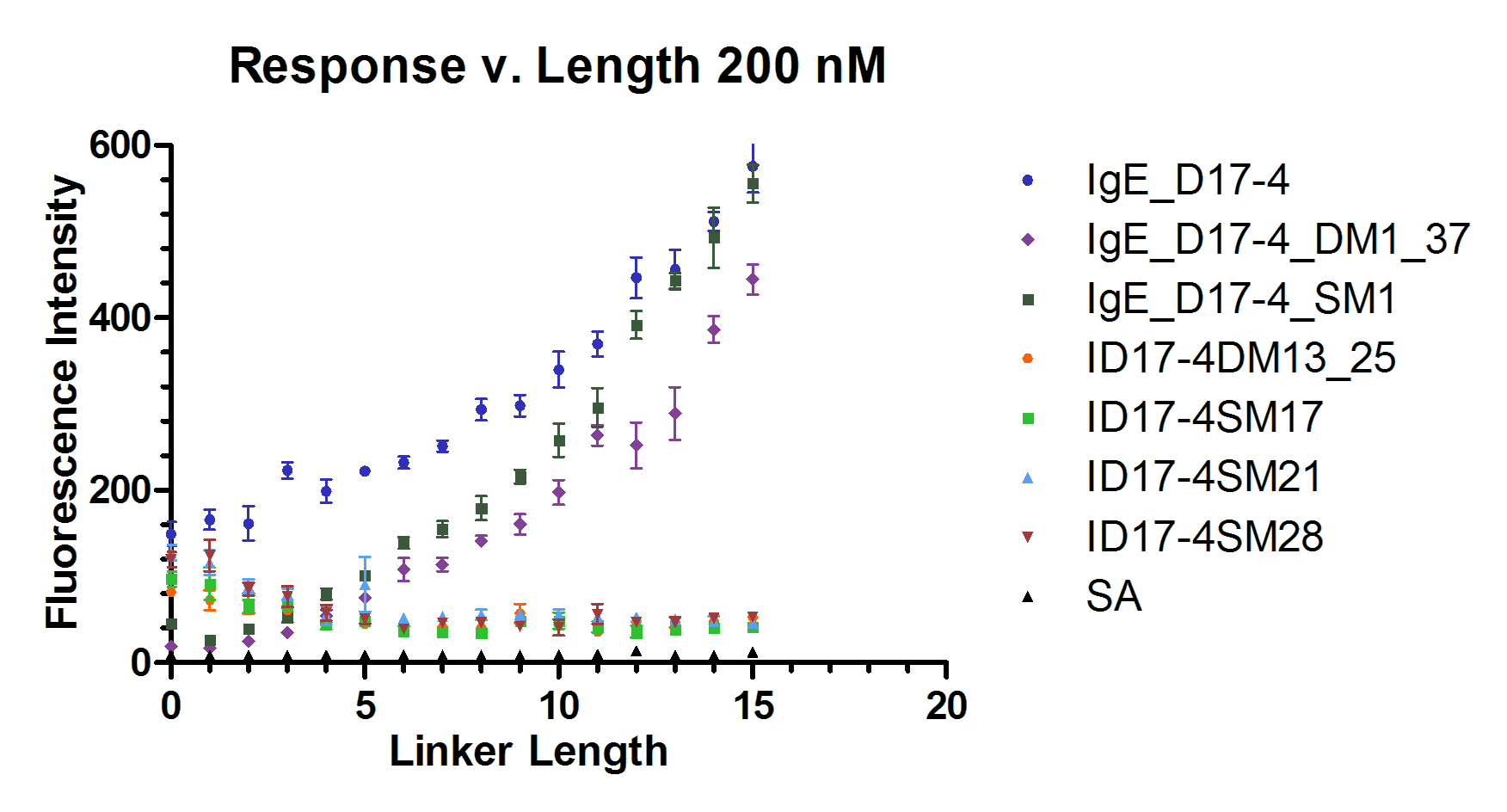


**Figure S2.** Response of different T linker sequences to 200 nM Cy3-IgE in PBSMTB buffer. Error bars represent SEM of raw fluorescence intensity values for 5-15 replicates of each sequence.


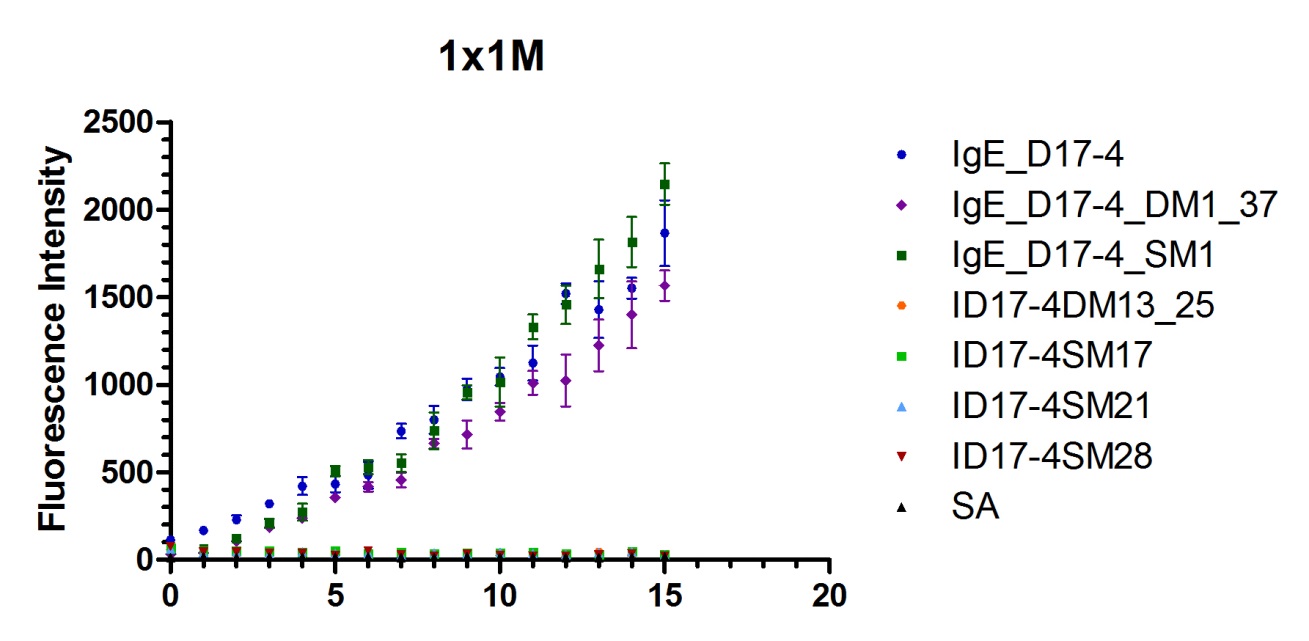


**Figure S3.** Response of different T linker sequences to 100 nM Cy3-IgE in PBSMTB buffer on 1X1M microarray. Error bars represent SEM of raw fluorescence intensity values for 5-15 replicates of each sequence.


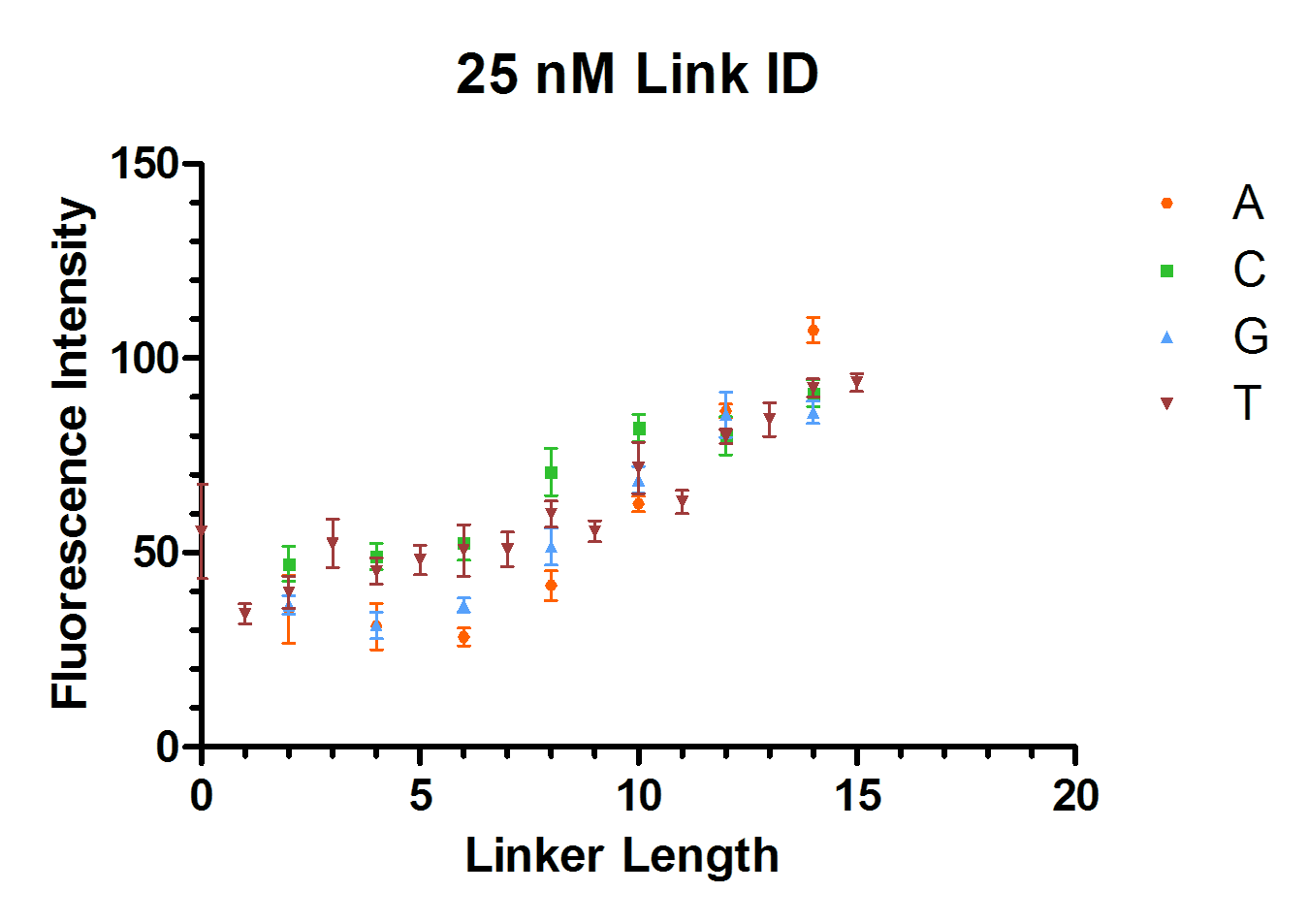


**Figure S4.** Response of IgE_D17-4 aptamer using different nucleobase linkers to 25 nM Cy3-IgE in PBSMTB buffer. Error bars represent SEM of raw fluorescence intensity values for six replicates of each sequence.


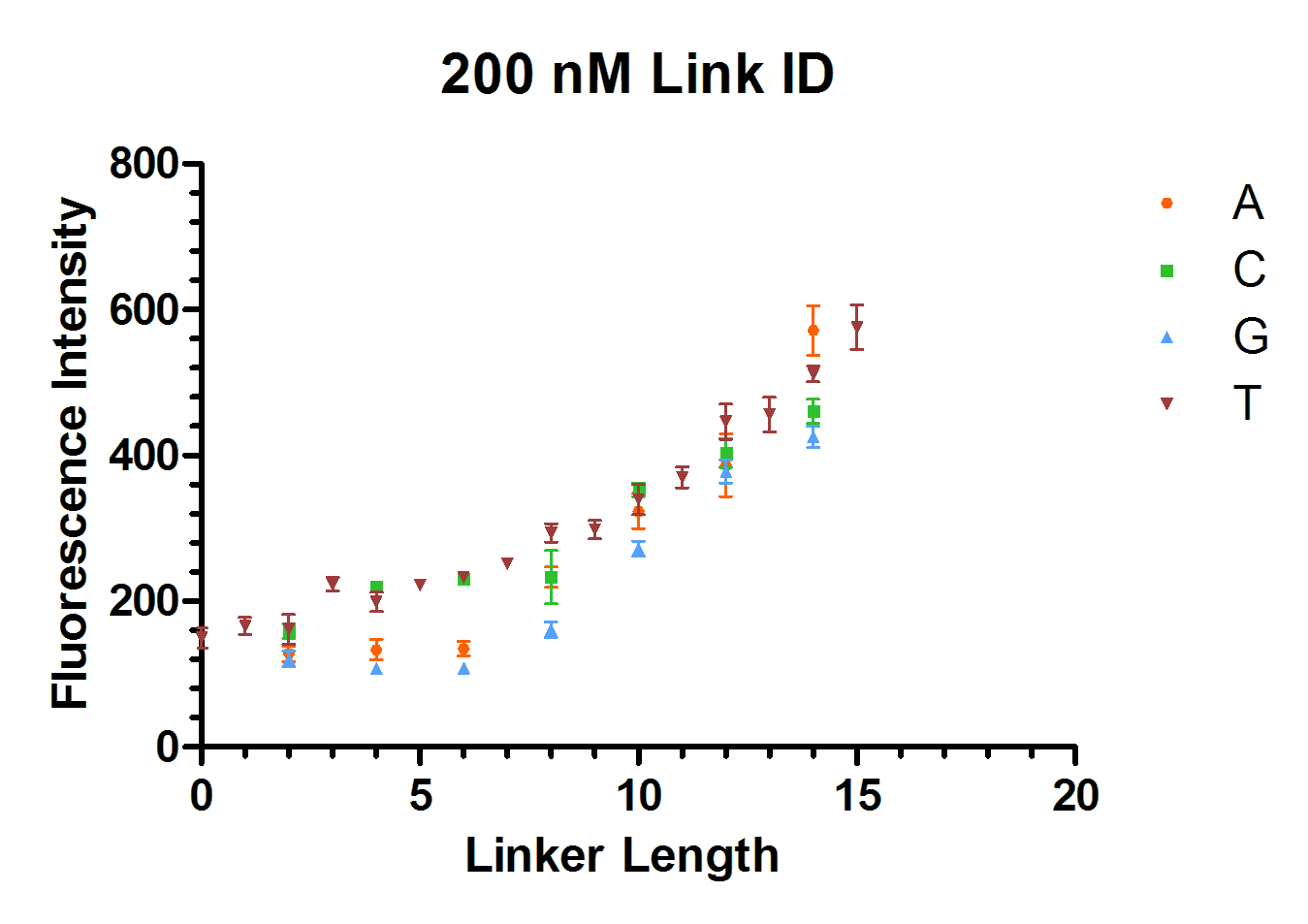


**Figure S5.** Response of IgE_D17-4 aptamer using different nucleobase linkers to 200 nM Cy3-IgE in PBSMTB buffer. Error bars represent SEM of raw fluorescence intensity values for six replicates of each sequence.


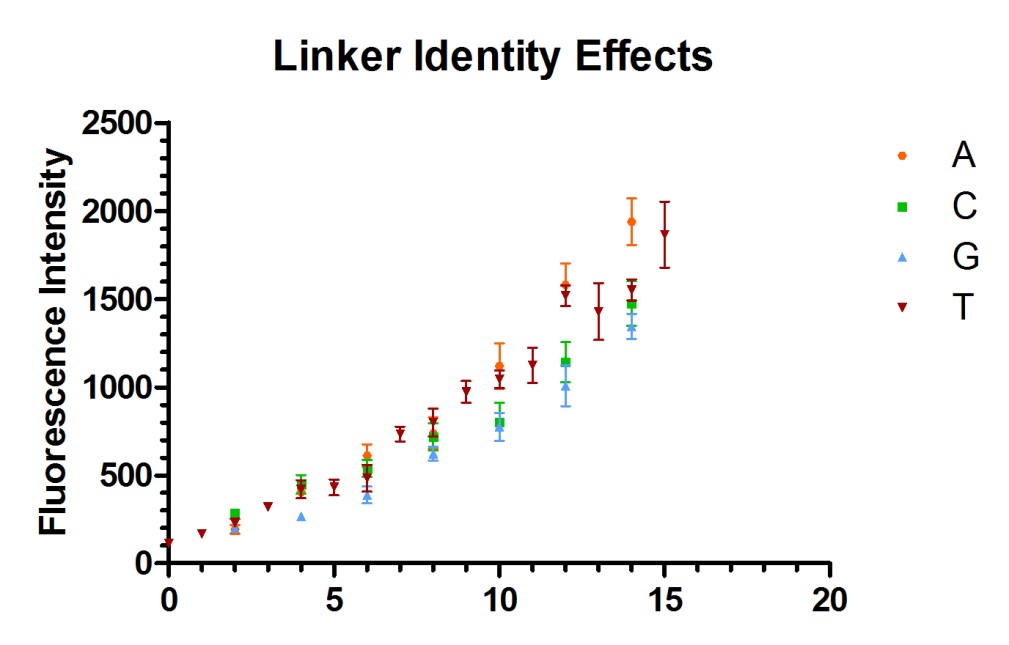


**Figure S6.** Response of IgE_D17-4 aptamer using different nucleobase linkers to 100 nM Cy3-IgE in PBSMTB using a 1X1M array. Error bars represent SEM of raw fluorescence intensity values for six replicates of each sequence.


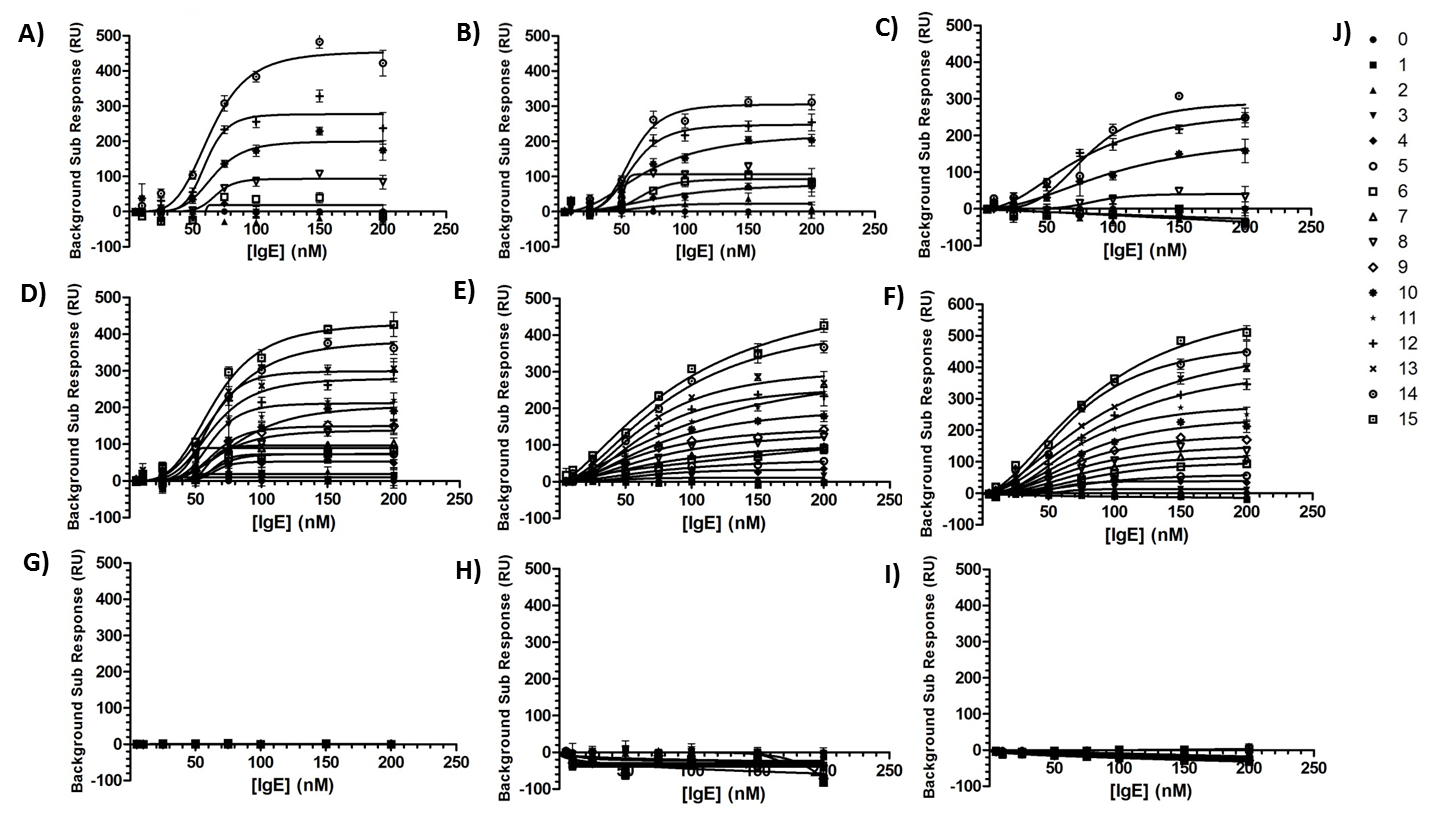


**Figure S7.** Background subtracted binding curves of Cy3-IgE to different sequences across various linker lengths. A) IgE_D17-4, A linker; B) IgE_D17-4, C linker; C) IgE_D17-4, G linker; D) IgE_D17-4, T linker; E) IgE_D17-4_DM1_37, T linker; F) IgE_D17-4_SM1, T linker; G) SA, T linker; H) ID17-4SM21, T linker; I)IgE_17-1, T linker; J) Legend for Figures S7A-I. Error bars designate SEM for six replicates.

K_d_ curves were established using the same methodology as the 8X15k Microarray Methodology described in the materials and methods section for 5-200 nM Cy3-IgE. The linker length=0 values for each concentration were subtracted from the other linker lengths to account for background. The background subtracted values for each sequence were plotted in GraphPad Prism to a one site- specific binding with Hill slope model. Average K_d_ for each sequence is reported as the average of all values at linker lengths= 10-15 ± the standard error of the K_d_’s for the same parameters. K_d_ for IgE_17-4 T linker lengths 10-15 were: 81.1 ± 5.5 nM, 61.9 ± 3.0 nM, 62.6 ± 5.5 nM, 60.2 ± 3.5 nM, 67.3 ± 3.3 nM, 64.6 ± 3.6 nM. All t-tests were performed in GraphPad Prism using a two-tailed t-test at 95% CI.


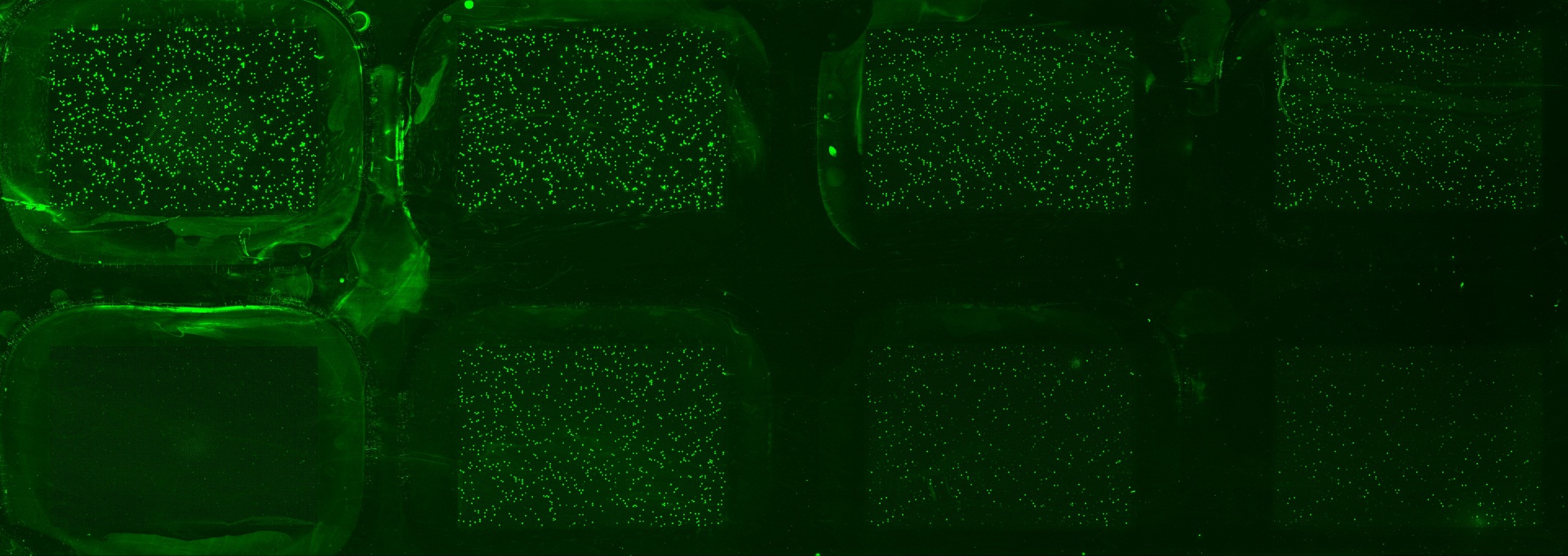

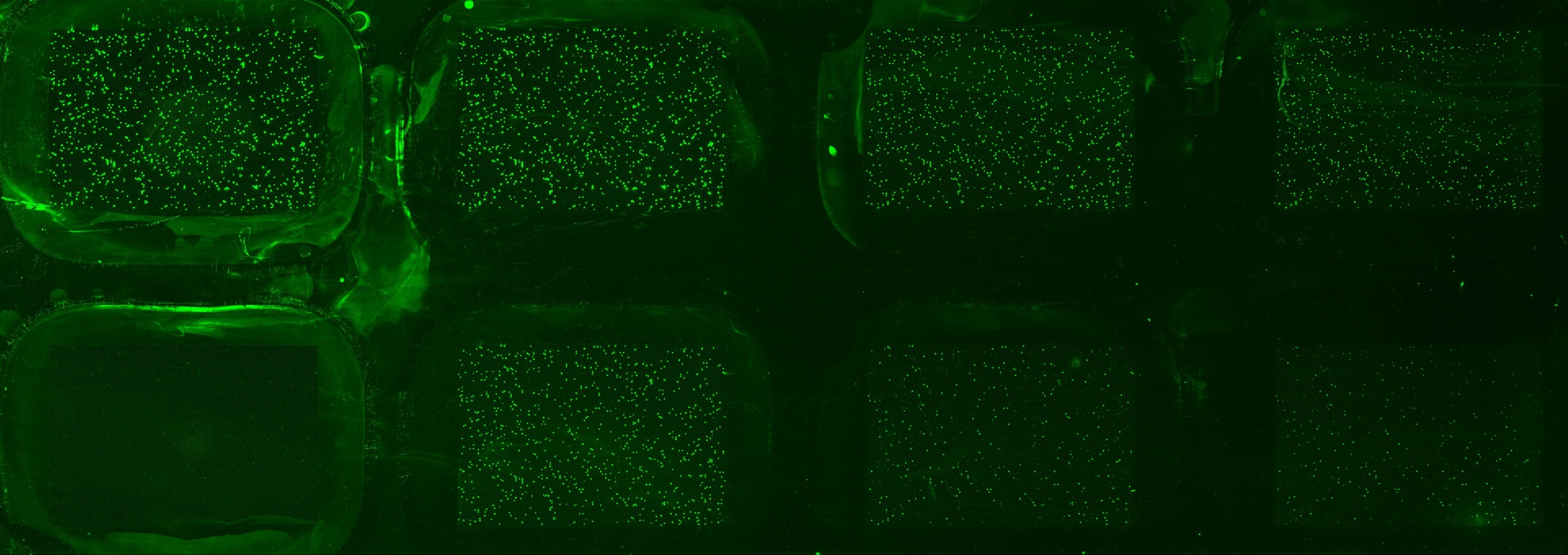


**Figure S8.** Image of one segment of an aptamer immobilized 8X15k microarray with Left) only 10 nM Cy3-Streptavidin; Right) 10 nM Biotin-IgE + 10 nM Cy3-Streptavidin. The images showed that fluorescence observed using the indirect binding method was a result of IgE binding rather than nonspecific Cy3 dye or streptavidin interactions.


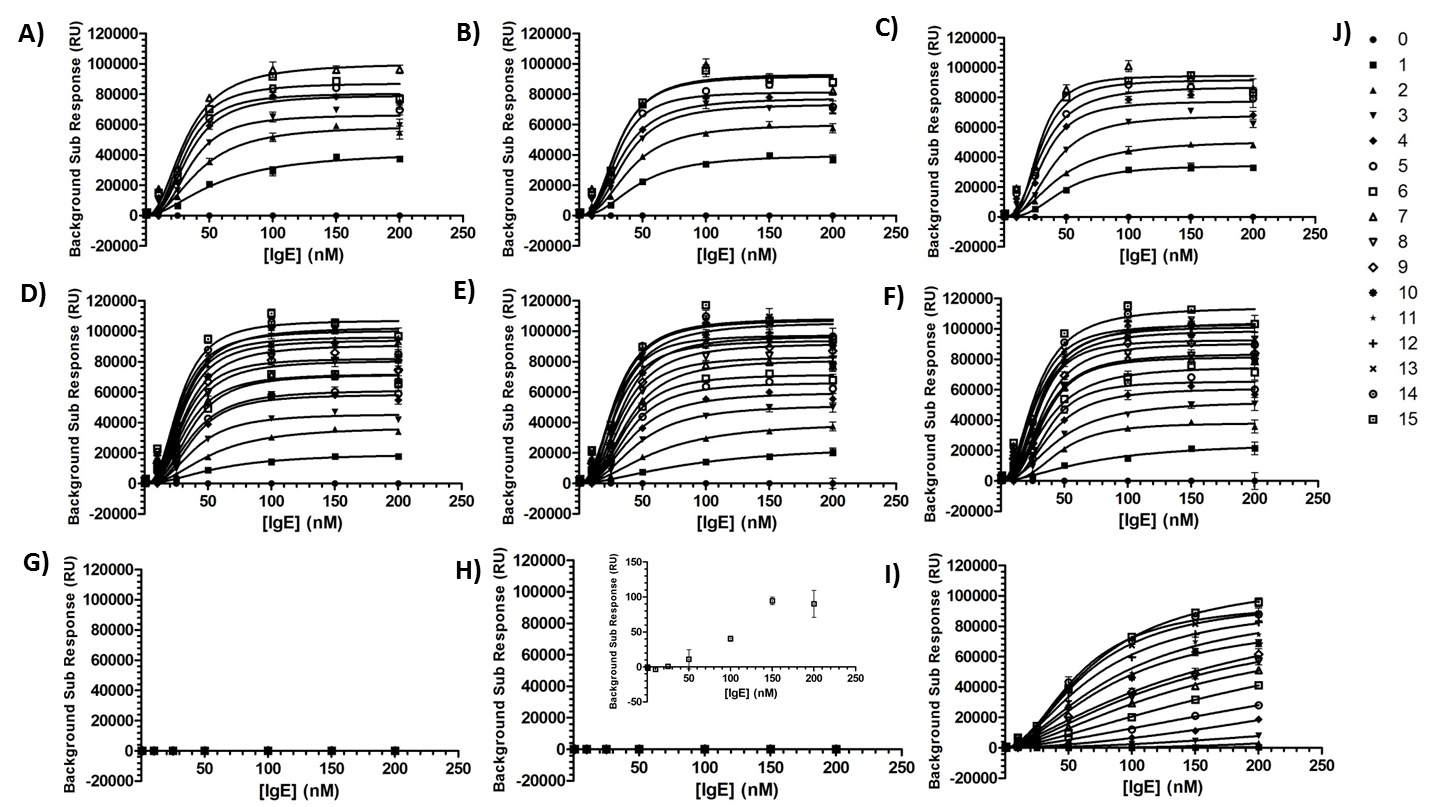


**Figure S9.** Background subtracted binding curves of biotin-IgE/Cy3-streptavidin to different sequences across various linker lengths. A) IgE_D17-4, A linker; B) IgE_D17-4, C linker; C) IgE_D17-4, G linker; D) IgE_D17-4, T linker; E) IgE_D17-4_DM1_37, T linker; F) IgE_D17-4_SM1, T linker; G) SA, T linker; H) ID17-4SM21, T linker; Inset: scaled ID17-4SM21 binding; I)IgE_17-1, T linker; J) Legend for Figures S7A-I. Error bars designate SEM for 5-15 replicates, [biotin-IgE]= 10 nM, [Cy3-SA]= 10 nM.


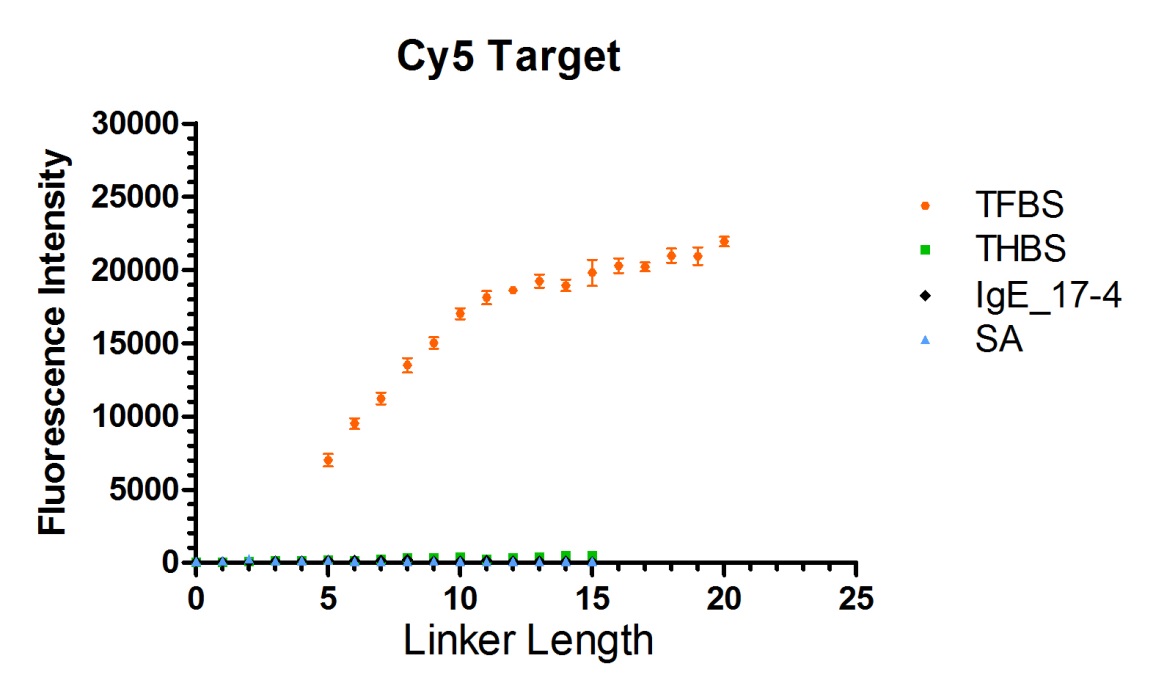


**Figure S10.** Binding of T-linker sequences to Cy5 dye in PBSMTB. Error bars designate SEM for 6 replicates.
